# Transplant Trial Watch

**DOI:** 10.3389/ti.2022.10652

**Published:** 2022-07-07

**Authors:** John M. O’Callaghan

**Affiliations:** ^1^ University Hospitals Coventry & Warwickshire, Coventry, United Kingdom; ^2^ Centre for Evidence in Transplantation, University of Oxford, Oxford, United Kingdom

**Keywords:** kidney transplantation, anti-inflammatory drugs, squamous cell carcinoma, topical sirolimus, randomised controlled trial

To keep the transplantation community informed about recently published level 1 evidence in organ transplantation ESOT and the Centre for Evidence in Transplantation have developed the Transplant Trial Watch. The Transplant Trial Watch is a monthly overview of 10 new randomised controlled trials (RCTs) and systematic reviews. This page of Transplant International offers commentaries on methodological issues and clinical implications on two articles of particular interest from the CET Transplant Trial Watch monthly selection. For all high quality evidence in solid organ transplantation, visit the Transplant Library: www.transplantlibrary.com.

Randomised Controlled Trial 1 Efficacy and Safety of Iguratimod Supplement to the Standard Immunosuppressive Regimen in Highly Mismatched Renal Transplant Recipients: A Pilot Study.

*by Tao, J., et al. Frontiers in Immunology 2021; 12: 738392.*

## Aims

This study aimed to assess the effect and safety of Iguratimod (IGU) combined with standard immunosuppressive regimen in highly HLA-mismatched kidney transplant patients.

## Interventions

Patients were randomised to either the IGU or non-IGU group.

## Participants

60 highly HLA-mismatched renal transplant recipients.

## Outcomes

The primary outcomes were biopsy-proven acute rejection and functional allograft survival. The secondary outcomes were the safety profile, donor-specific antibody (DSA) and other indicators.

## Follow-Up

52 weeks.

## CET Conclusion

This small pilot RCT investigated whether the addition of the disease-modifying anti-rheumatoid drug (DMARD) Iguratimod (IGU) can improve outcomes in poorly-mismatched renal transplant recipients. The study itself was unblinded, but nephrologists scoring protocol biopsies were blinded to treatment allocation. Both modified intent-to-treat and per-protocol analyses are reported. Patients receiving IGU had numerically lower incidence of biopsy-proven acute rejection, although not achieving statistical significance due to the small sample size. The results presented do show some promise for the use of IGU following renal transplantation, but larger studies will be required to confirm any benefit. It should be noted that the baseline rate for biopsy-proven acute rejection was relatively high for a Tac/MMF/Pred based regimen (29.6%). Another potential limitation is that patients were only eligible at least 2 weeks post-transplant—anti-inflammatory drugs of this nature may be most effective if given from the day of transplant.

## Jadad Score

3.

## Data Analysis

Modified intention-to-treat analysis.

## Allocation Concealment

No.

## Trial Registration

ClinicalTrials.gov—NCT02839941.

## Funding Source

Non-industry funded.

Randomised Controlled Trial 2 Chemoprevention of Cutaneous Squamous Cell Carcinoma and Its Precursors in Solid Organ Transplant Recipients Using Topical Sirolimus: A Randomized, Double-Blind, Placebo-Controlled Pilot Trial.

*by Chong, S., et al. Journal of the American Academy of Dermatology 2022 [Online ahead of print].*

## Aims

This study aimed to examine whether topical application of sirolimus could safely reduce the incidence of keratinocyte cancer (KC) in solid organ transplant recipients.

## Interventions

Forearms of patients were randomised to either receive topical sirolimus or placebo.

## Participants

29 adult solid organ transplant recipients with a history of basal cell carcinomas or squamous cell carcinomas (SCC) as well as keratotic lesions on the back of the forearms and hands.

## Outcomes

Number of keratotic lesions, change in keratotic lesions, number of intraepidermal carcinoma, and number of SCC.

## Follow-Up

24 months.

## CET Conclusion

This small, blinded pilot study randomised transplant recipients with a history of basal and squamous cell carcinoma (BCC/SCC) to apply topical sirolimus to one forearm/hand and placebo to the other for 12 weeks. There was a significant reduction in the risk of keratotic lesions in the sirolimus group at 12 weeks, which resulted in a significant reduction in the risk of intraepithelial carcinomas at 24 months (4 in the sirolimus arm vs. 12 in the placebo arm). Whilst clearly small and underpowered for firm conclusions, this pilot study does provide some evidence of efficacy and feasibility in support of a larger efficacy study.

## Trial Registration

ACTRN12618001961235.

## Funding Source

Non-industry funded.

## Clinical Impact Summary

This is a short letter in the Journal of the American Academy of Dermatology, yet it shows some interesting and clear-cut results from a study with elegant design.

Transplant recipients who were high risk for skin lesions, having at least five Basal Cell Carcinoma (BCC) or Squamous Cell Carcinoma (SCC) in the last 5 years, and at least 5 current keratotic lesions on the back of the forearms or hands, were selected. By randomising one hand/forearm from each patient to treatment and the other hand/forearm to placebo, the study introduced a paired design that not only effectively doubled the recruitment number but also reduced differences between study “arms” (no pun intended). The blinding was achieved by daily application of two topical preparations that were physically indistinguishable, one with additional 1% sirolimus.

At 12 weeks, 18 patients completed the regimen. 11 patients stopped applying the study preparations but had an average of 5 weeks of application and only 1 stopped due to contact dermatitis. The number of keratotic lesions was significantly reduced in each patient on the treated hand/forearm (31%) but was increased in the control hand/forearm (6%). Over 24 months of follow up there were 3-times fewer intraepithelial carcinomas on the treated hand/forearm, using intention to treat analysis (There was no significant difference in the SCC numbers).

The preparation of topical sirolimus appears to be quite effective at preventing intraepithelial carcinoma on the upper limbs of high-risk transplant patients. It would be good now to see longer term follow up to see how the effect is maintained.

